# Supplementary material for: MetaRibo-Seq measures translation in microbiomes
Source: Nat Commun. 2020 Jun 29;11:3268. doi: 10.1038/s41467-020-17081-z (PMC7324362; doi:10.1038/s41467-020-17081-z)
Supplement: Supplementary file 10 — Supplementary Data 7 [file 41467_2020_17081_MOESM10_ESM.zip › File2/Confidence_High_Taxonomy/421031_out.krona.html]

Javascript must be enabled to view this page.

members
magnitude
magnitudeUnassigned
count
unassigned
taxon
rank

421031\_out

85

85
2
superkingdom

phylum
976
85

200643
85
class

85
171549
order

family
85
815

816
85
genus

1
28116

SRS147377\_contig\_number\_10141
species

species

SRS098644\_contig\_number\_22995SRS098881\_contig\_number\_15230SRS143417\_contig\_number\_28718
3
817

species
29523
1

SRS147271\_contig\_number\_14571

1
820

SRS893327\_contig\_number\_823
species

species

SRS017745\_contig\_number\_1507
46506
1

species

SRS020328\_contig\_number\_38104
1
818

species
821
73

SRS011239\_contig\_number\_26480SRS012902\_contig\_number\_contig-100\_916.15431SRS012969\_contig\_number\_17050SRS013382\_contig\_number\_2293SRS013638\_contig\_number\_11933SRS014459\_contig\_number\_12718SRS014948\_contig\_number\_13311SRS015065\_contig\_number\_31166SRS015431\_contig\_number\_76900SRS015486\_contig\_number\_1779SRS015694\_contig\_number\_12922SRS016056\_contig\_number\_7758SRS016629\_contig\_number\_2623SRS017375\_contig\_number\_154SRS017701\_contig\_number\_8164SRS017859\_contig\_number\_1994SRS018623\_contig\_number\_4145SRS018817\_contig\_number\_contig-100\_445.131442SRS018888\_contig\_number\_3413SRS019693\_contig\_number\_8502SRS021153\_contig\_number\_674SRS023829\_contig\_number\_contig-100\_833.12597SRS024492\_contig\_number\_8706SRS042966\_contig\_number\_18876SRS045004\_contig\_number\_7807SRS045645\_contig\_number\_31267SRS050520\_contig\_number\_1157SRS050998\_contig\_number\_16649SRS053087\_contig\_number\_2589SRS053335\_contig\_number\_18108SRS053649\_contig\_number\_11265SRS054905\_contig\_number\_15157SRS055966\_contig\_number\_367SRS062654\_contig\_number\_14835SRS062701\_contig\_number\_1366SRS063127\_contig\_number\_1199SRS063370\_contig\_number\_10704SRS065397\_contig\_number\_contig-100\_452.82179SRS074964\_contig\_number\_6067SRS075398\_contig\_number\_contig-100\_439.21200SRS077552\_contig\_number\_14940SRS077641\_contig\_number\_contig-100\_42.130118SRS077991\_contig\_number\_171SRS097958\_contig\_number\_885SRS101376\_contig\_number\_26728SRS104084\_contig\_number\_13891SRS1041092\_contig\_number\_9811SRS1041118\_contig\_number\_11354SRS1041141\_contig\_number\_4306SRS1041142\_contig\_number\_3595SRS1041143\_contig\_number\_4919SRS1054716\_contig\_number\_10344SRS1055022\_contig\_number\_19719SRS1055038\_contig\_number\_6335SRS142923\_contig\_number\_20052SRS143722\_contig\_number\_4476SRS144297\_contig\_number\_2519SRS144362\_contig\_number\_10988SRS144506\_contig\_number\_20781SRS144714\_contig\_number\_23476SRS146813\_contig\_number\_7758SRS147022\_contig\_number\_1760SRS147039\_contig\_number\_1943SRS147766\_contig\_number\_18285SRS148424\_contig\_number\_contig-100\_396.168987SRS149075\_contig\_number\_10701SRS893172\_contig\_number\_13645SRS893187\_contig\_number\_16889SRS893259\_contig\_number\_1149SRS893288\_contig\_number\_1213SRS893341\_contig\_number\_6250SRS893383\_contig\_number\_31509SRS971275\_contig\_number\_27469

1
2292944

SRS143991\_contig\_number\_17142
species

species
1
47678

SRS014412\_contig\_number\_13083

species
2
291644

SRS050422\_contig\_number\_12625SRS144537\_contig\_number\_41857
